# Poster Session II - A306 A SYSTEMATIC REVIEW OF THE EFFECTIVENESS AND SAFETY OF SEQUENTIAL RESCUE THERAPY WITH JAK INHIBITORS IN ACUTE SEVERE ULCERATIVE COLITIS

**DOI:** 10.1093/jcag/gwaf042.306

**Published:** 2026-02-13

**Authors:** A Cintosun, J Liu, N S Rai, N Narula

**Affiliations:** McMaster University, Hamilton, ON, Canada; McMaster University, Hamilton, ON, Canada; McMaster University, Hamilton, ON, Canada; McMaster University, Hamilton, ON, Canada

## Abstract

**Background:**

Acute severe ulcerative colitis (ASUC) affects 25% of patients with ulcerative colitis and can result in colectomy. The standard of care is to treat with intravenous steroids, with failure followed by rescue therapy such as infliximab (IFX) or cyclosporine (CsA), and colectomy if rescue therapy is unsuccessful. Janus kinase inhibitors (JAKi) are small molecule medications with rapid onset which have been employed for ASUC, but the safety and efficacy of their use after failure of IFX or CsA requires further study.

**Aims:**

To determine the safety and efficacy of using JAKi salvage therapy for patients with ASUC who have failed rescue therapy with IFX or a calcineurin inhibitor.

**Methods:**

We performed a systematic review of MEDLINE, Embase, and PubMed. Two authors independently screened studies for inclusion based on ASUC, steroid failure, and treatment with at least two rescue therapies (IFX or a calcineurin inhibitor followed by a JAKi) within three months.

**Results:**

Twelve studies with 58 patients (22% female) were included (4 retrospective cohort studies, 8 case series or reports). All but one paediatric study were conducted in adults. Follow-up ranged from six weeks to 18 months.

Following IV steroids, patients received two or three rescue therapies. Fifty-five patients were treated with IFX followed by JAKi (tofacitinib or upadacitinib). The remaining three received either 1) IFX then CsA then JAKi; 2) IFX then combination CsA and JAKi; or 3) tacrolimus then JAKi.

Tofacitinib induction dosing was either 10 mg twice daily (BID), thrice daily (TID), or TID for a few days followed by BID. Upadacitinib induction was 45 mg daily.

Clinical response to sequential rescue therapy with a JAKi occurred in 45 (78%) patients ranging from 2 to 18 days. Clinical remission was reported in 33 patients (57%) ranging from three weeks to one year. The three-month colectomy rate was 24% (n = 14). Four studies reported longer-term colectomy data: two additional patients required colectomy by twelve months.

Four of 58 patients had one minor adverse event (skin lesion, arthralgia or myalgia, cardiovascular event, or not specifically identified). There were no serious adverse events or mortality reported.

**Conclusions:**

This is the first systematic review of sequential rescue therapy including JAKi in ASUC. In patients who failed rescue therapy with IFX or a calcineurin inhibitor, sequential rescue therapy with JAKi was safe and led to clinical response and avoidance of colectomy in a substantial proportion of patients.

**Funding Agencies:**

None

